# Inoculation of heavy metal resistant bacteria alleviated heavy metal-induced oxidative stress biomarkers in spinach (*Spinacia oleracea* L.)

**DOI:** 10.1186/s12870-024-04757-7

**Published:** 2024-03-27

**Authors:** Muhammad Jamil, Ijaz Malook, Shafiq Ur Rehman, Muhammad Mudasar Aslam, Muhammad Fayyaz, Gulmeena Shah, Alevcan Kaplan, Muhammad Nauman Khan, Baber Ali, Rana Roy, Sezai Ercisli, Steve Harakeh, Mohammed Moulay, Muhammad Ammar Javed, Amany H. A. Abeed

**Affiliations:** 1 Department of Biotechnology and Genetic Engineering, Kohat University of Science & Technology (KUST), Kohat, 26000 Pakistan; 2 Department of Biology, University of Haripur, Haripur, 22620 Pakistan; 3 Department of Botany, University of Science and Technology, Bannu, 28100 Pakistan; 4 Institute of Crop Science, College of Agriculture and Biotechnology, Zhejiang University, Hangzhou, 310027 China; 5 Department of Crop and Animal Production, Sason Vocational School, Batman University, Batman, 72060 Turkey; 6 Department of Botany, Islamia College Peshawar, Peshawar, 25120 Pakistan; 7 University Public School, University of Peshawar, Peshawar, 25120 Pakistan; 8 Department of Plant Sciences, Quaid-I-Azam University, Islamabad, 45320 Pakistan; 9 Institute of Plant Nutrition and Soil Science, Christian-Albrechts-Universität Zu Kiel, 24118 Kiel, Germany; 10 Department of Agroforestry & Environmental Science, Sylhet Agricultural University, Sylhet, 3100 Bangladesh; 11 Department of Horticulture, Agricultural Faculty, Ataturk University, Erzurum, 25240 Türkiye; 12 HGF Agro, Ata Teknokent, TR-25240 Erzurum, Türkiye; 13 King Fahd Medical Research Centre, King Abdulaziz University, Jeddah, 21589 Saudi Arabia; 14 Faculty of Medicine, Yousef Abdul Latif Jameel Scientific Chair of Prophetic Medicine Application, King Abdulaziz University, Jeddah, 21589 Saudi Arabia; 15 Stem Cell Research Unit, King Fahd Medical Research Center, King Abdulaziz University, 21589 Jeddah, Saudi Arabia; 16 Department of Medical Laboratory Sciences, Faculty of Applied Medical Sciences, King Abdulaziz University, Jeddah, 21589 Saudi Arabia; 17 Institute of Industrial Biotechnology, Government College University, Lahore, 54000 Pakistan; 18 Department of Botany and Microbiology, Faculty of Science, Assiut University, Assiut, 71516 Egypt

**Keywords:** Antioxidative metabolism, Heavy metals toxicity, Metallothioneins (MTs), Remediation, Scanning electron microscope (SEM)

## Abstract

Most vegetable crops are severely affected by the uptake of heavy metals from the soil. Heavy metals in vegetable bodies generate reactive oxygen species (ROS) that unbalance the antioxidant defense system. This study was initiated to determine the physiological and biochemical characteristics of spinach plants grown on soil contaminated with heavy metals and responding to *Bacillus cereus* and *Bacillus aerius* were isolated from soil contaminated with heavy metals. Heavy metal contamination led to a significant reduction in seed germination, seedling biomass, protein, and total nitrogen content of spinach plants grown in contaminated soils compared to control soils. In contrast, a significant increase in the content of metallothioneins and antioxidant enzymes was observed. Plants inoculated with *B. cereus* and *B. aerius* significantly reduced the oxidative stress induced by heavy metals by improving seed germination (%), seedling growth, nitrogen, and protein content. The content of metallothioneins and the activities of antioxidant enzymes were reduced in spinach plants grown from seeds inoculated with bacterial strains. In addition, plants inoculated with, *B. cereus* and *B. aerius* showed greater stomata opening than plants grown on soil contaminated with heavy metals, whose stomata were almost closed. These results suggested that both bacterial strains enhanced plant growth by reducing oxidative stress caused by metals.

**Supplementary Information:**

The online version contains supplementary material available at 10.1186/s12870-024-04757-7.

## Introduction

Metal pollution has a detrimental effect on the soil ecosystem, leading to various ecological changes, such as changes in soil structure, reduction in soil fertility and effects on soil microorganisms [1]. Plants growing in polluted soils take up toxic metals that interfere with plant growth. Morphological, biochemical and physiological growth processes in plants are significantly altered by the toxicity of heavy metals [2–4]. Plants synthesis active compounds known as reactive oxygen species (ROS) as a result of heavy metal toxicity [5–7]. ROS induce oxidative stress in plants, which affects growth characteristics and alters the redox status of plant cells [8–10]. Vegetables grown near industrial sites develop poorly due to the large amount of heavy metals in the soil [11]. Microorganisms play a key role in promoting plant health [12]. Plant growth-promoting rhizobacteria (PGPR) are a collection of free-living rhizobacterial communities that competitively colonise root surfaces and promote plant growth by secreting a variety of phytostimulantchemicals and sustainably prevent various causes of host diseases [13–15]. Plants are further protected from the invasion of phytopathogens by PGPR that secrete antibiotics, antifungal chemicals, hydrocyanic acid (HCN), chitinase, and other substances. These PGPR strains have been detected for several years in the metal-contaminated rhizospheres of various crops, including vegetables [16]. Plant growth-promoting rhizobacteria (PGPR) can enhance soil productivity and bioremediation efficacy by utilising various microbes and chemicals to treat or detoxify contaminants in an environmentally benign manner [17]. These PGPR and their exudates detoxify a wide range of organic and inorganic pollutants such as heavy metals and various pesticides and herbicides [18]. PGPRs are most commonly used for this purpose as they can be used in situ and are eco-friendly and non-polluting environmentally favourable [19, 20]. PGPRs improve plant growth and production by providing plants and soils with the necessary nutrients and bioremediate polluted soils [21]. The rapidly expanding industry, uncontrolled and untreated release of xenobiotic pollutants and the use of low-quality liquids (wastewater) for irrigation in agriculture pose a serious and unsustainable threat to the sustainability of agroecological niches [22–24]. On the other hand the availability of metals to plants, is determined by soil variables such as pH, cation exchange capacity (CEC), organic matter content and clay adsorption [25]. Heavy metals accumulate in the soil and enter the food chain, where they are passed on to end consumers and endanger human health [26]. Moreover, the toxicity of heavy metals entering plant tissue can interfere with a number of physiological activities. The toxicity of heavy metals also leads to oxidative stress, disruption of pigment function and changes in protein activity [27]. Under metal stress, excessive ROS production can cause severe damage to plant cell structures, including (i) oxidation of proteins and lipids, (ii) nucleic acid damage, (iii) enzyme inhibition, and (iv) cell death [23]. Plants have usually developed various adaptations to protect themselves from the harmful effects of ROS [20]. Plants have developed different approaches against the toxicity of heavy metal ions to minimise their harmful effects. Plant root cells adsorb heavy metals through the formation of polysaccharide complexes [28] or the binding of apoplasts with organic acid [29, 30]. It is also possible that heavy metals are stored in the cell vacuoles [31], metallothioneins (MTs) and phytochelatins (PCs) are produced like metal-binding compounds [32] and glutathione also detoxifies ROS [33]. The plants activated various antioxidant enzymes thatprotected the plants by reducing oxidative stress [34]. In addition to these plant defence strategies against heavy metals, soil microorganisms, especially bacteria play an important role in plant growth through the uptake of various nutrients and protection against various diseases [35]. Soil microbiota has the ability to detoxify heavy metals and utilise them for beneficial purposes in heavy metal polluted environments [36]. Ramaiah and Vardanyan [37], in their study to evaluate the detoxification potential of cadmium and lead, investigated the bacteria, *Alcaligenes faecalis*, *Bacillus pumilus*, *Pseudomonas aeruginosa*, and *Brevibacterium iodinium*, which are highly resistant to mercury and can grow at 25 ppm or higher mercury concentrations, in a growth medium of 100 ppm and 72 ppm, respectively. They wereobserved to remove more than 70% of Cd and 98% of Pb within 96 h. Shahraki et al. [38] found that *Pseudomonas fluorescens* and *B. cereus* strains had the greatest effect on lead assimilation at 2175 and 1862 ppm, respectively. Bilal et al. [39] found that co- refinement of LHL10 and LHL06 promoted plant growth characteristics and photosynthetic activity, glutathione, catalase and superoxide dismutase activities, and decreased lipid peroxidation by increasing macronutrient uptake under high temperature and drought stress. Gao et al. [40] found that under heavy metal stress, inoculation with immobilised bacteria significantly promoted the growth of alfalfa, with the dry weight of roots, stems and leaves increasing by 19.8%, 6.89% and 14.6%, respectively. Microbes, especially bacteria, have evolved various mechanisms to cope with heavy metal stress in anthropogenically contaminated media and promote plant growth [41]. Bacteria are known to have evolved a variety of mechanisms to develop resistance to heavy metals, including: Expulsion of metal through a permeable barrier, removal of metal from cells by active transport, intracellular physical sequestration of metal by proteins or other ligands to protect metal-sensitive cellular targets from damage, extracellular sequestration, transformation and detoxification [42, 43].

Spinach (*Spinacia oleracea* L.) is a plant food grown mainly in semi urban areas of the world, which are affected by irrigation with effluents containing heavy metals from the industrial sector [44, 45]. Compared to fruits and root vegetables, spinach has the potential to absorb greater amounts of heavy metals and toxic elements from the rhizosphere and convert them into edible parts [46, 47]. Our previous study has shown that soils contaminated with heavy metals negatively affect the growth of spinach due to high lipid peroxidation [44]. Scientists use various physicochemical and biological approaches to attenuate the oxidative stress induced by heavy metals,but the use of heavy metal resistant bacterial strains is very rare in this field. Against this background, the present work aims to investigate the physiological and biochemical effects of *B. aerius* and *B. cereus* strains on spinach grown in soils contaminated with heavy metals. The novelty of this work lies in the innovative technique of using heavy metal resistant bacteria to improve the indicators of oxidative stress caused by heavy metals in spinach (*Spinacia oleracea* L.). By utilising the unique properties of bacteria to reduce the negative effects of heavy metal contamination on plant physiology, this strategy represents a breakthrough in sustainable agriculture. The study not only addresses the environmental problems associated with heavy metal pollution, but also offers a promising and environmentally benign strategy to increase the resilience of plants to the harmful effects of heavy metals. The inclusion of microbial interventions to mitigate stress in plants offers a new dimension to agricultural practises and highlights the potential for more environmentally conscious and sustainable food production.

## Materials and methods

### Materials and reagents

LB media (Sigma-Aldrich, Germany), CuSO_4_ (Sigma-Aldrich, Germany, > 98%), K_2_SO_4_ (Applychem, Germany, 99%), FeSO_4_ (Duskan Pure chemicals, Korea, 98%)_,_ H_2_SO_4_ (V.S Chem house, Thiland, 97.5%)_,_ NaOH (Sigma-Aldrich, germany, 98%), EDTA (Applychem, Germany, 99%), Tris–HCl (Solar Bioscience & Tech, China, 99.5%), Nitrobenzoic acid (BDH Labortery Supply, England, 98%), phosphate buffer, Coomassie Brilliant Blue (Applychem, Germany), Protein molecular marker (Benchmark Protein Ladder), Bovine serum albumin (Sigma-Aldrich, Germany, 98%), Nitro blue tetrazolium(Malford, UK) Riboflavin (Daejung Chemical, Korea, 98%) H_2_O_2_ (VWR Chemical, Balgium, 30%), Guaiacol (Unichem chemical, 99%), Ascorbic acid (BDH Labortery Supply, England, 99%), RNA standard isolation kit (NucleoSpin RNA plant, Germany), cDNA synthesis kit (Thermo scientific RevertAid First strand, USA).

### Physiological parameters

Spinach (*Spinacia oleracea* L.) seeds (cv. Local Sindhi) seeds were obtained from National Agriculture Research Center (NARC) Islamabad, Pakistan and sterilized with 3% (v/v) NaOCl solution. After sterilization, the seeds were washed with deionized water to remove the residual NaOCl solution [48]. The soil contaminated with heavy metals was collected from agricultural fields irrigated with contaminated water in Hayatabad Industrial Estate Peshawar (HIEP) and Gadoon Industrial Estate Swabi (GIES), Khyber Pukhtoonkhwa, Pakistan. The collected contaminated soil was analyzed for heavy metals using standard protocols [49, 50]. Bacteria were isolated from the soils of HIEP and GIES and identified as *Bacillus aerius* and *Bacillus cereus* [51]. These strains were further cultured in for 24 h at 37 °C in LB media. The seeds were biologically-primed for 10 h in LB media supplemented with 2% sucrose to allow the bacterial strains to adhere with a cell suspension of 10^8^–10^9^ CFU/mL.

### Analysis of contaminated soil

In this experiment, soil samples were used. To purify soil samples, they were treated and autoclaved. Soil analyses were performed, including pH, texture, and EC. Soil organic matter was assessed using the procedures described in [52]. 2 g of soil was placed in a 500 mL conical flask using this procedure. The flask was then filled with 200 mL of distilled water, 10 mL of 1 N k_2_Cr_2_O_7_, and 10 mL of orthophosphoric acid. 30 drops of Diphenylamine (used as an indicator) were added to the mixture after half an hour. The reaction came to an end when a green colour appeared. The soil metal analysis was carried out using the method outlined by the authors of [53].

Seeds primed with bacterial strains were grown under greenhouse conditions in plastic pots containing 1.5 kg of autoclaved soil. The experiment was divided into nine treatments, with the control soil coming from well irrigated agricultural fields and the soil from contaminated agriculture fields irrigated with the polluted water from HIEP and GIES. The seeds were bio-coated with microbes and planted in plastic pots with uniform soil. Seed germination was recorded every 24 h. Plants were irrigated regularly as needed and harvested after 30 days to examine various physiological and biochemical growth characteristics.

### Biochemical parameters

#### Determination of total nitrogen and protein contents

The micro-Kjeldahl method was used to determine the total nitrogen and protein content [54]. A total of 1 g of plant material was placed in digestion tube consisting of CuSO_4_, K_2_SO_4_ and FeSO_4_ and 10 mL of concentrated H_2_SO_4_ solution. The mixture solution was heated in the digestion unit to completely homogenise it. After digestion and cooling, 20 mL of distilled water and then 10 mL of 50% NaOH solution were added to the mixture. To the mixture, 4% boric acid (50 mL) and methyl red indicator were added to complete the distillation process. The mixture was titrated against known N H_2_SO_4_ solution. The total nitrogen and protein content was determined according to the given formula [55].

$$\mathrm{Total}\;\mathrm{Protein}(\frac{\textrm{g}}{\text{g}})=\frac{\mathrm{Sample}\;\mathrm{Volume}-\mathrm{Blank}\;\mathrm{Volume}\;\mathrm {x}\;0.1\text{N}}{\mathrm{Dry}\;\mathrm{Weight}\;\mathrm{of}\;\mathrm{Sample}}\mathrm{x}\;1.4007$$

$$\mathrm{Total}\;\mathrm{Organic}\;\mathrm{Nitrogen}(\frac{\text{g}}{\textrm{g}})=\frac{\mathrm{Sample}\;\mathrm{Volume}-\mathrm{Blank}\;\mathrm{Volume}\;\mathrm{x}\;0.1\text{N}}{\mathrm{Dry}\;\mathrm{Weight}\;\mathrm{of}\;\mathrm{Sample}}\mathrm{x}\;6,25$$

where; Nitrogen factor = 1.4007 and Protein factor = 6.25.

#### Quantification of low molecular weight polypeptides of metallothioneins (MTs)

The metallothionein content was determined according to a standard protocol using the Ellmans reagent [56]. The plant samples were completely crushed and homogenized in a buffer solution for the reduction of protein disulfide bonds. The mixture was centrifuged at 10,000 rpm for 30 min to obtain MTs supernatants. The supernatants containing MTs were centrifuged at 6000 rpm for 10 min after addition of 1 mL of chilled ethanol 80 μL of chloroform and stored at low temperature for 60 min. For quantification of MTs, the supernatant was centrifuged again at 6000 rpm for 10 min. The collected pellets were resuspended in 1 mM EDTA and 100 μL 5 mM Tris–HCl at pH 7. The MTs mixture was stored at 25 °C for 30 min after addition of 420 μL 0.4 mM nitrobenzoic acid (pH 8) and 0.2 M phosphate buffer. The MTs content was determined at a wavelength of 412 nm wavelength using GSH as a standard solution.

The MTs proteins were extracted using protein extraction buffer previously used previous procedure [57]. The Bradford method was used to quantify the proteins before separation by SDS-PAGE using 30 g protein per lane according to the protocol [58]. Proteins were separated using a mini gel electrophoresis unit (USA) on a 17% SDS gel at 80 V for 150 min. Coomassie Brilliant Blue R-250 (Sigma) was used for gel staining. Standard protein molecular weight markers (Benchmark Protein Ladder) were used to compare MTs protein subunits in the electrophorogram [59].

#### Protein and antioxidant enzyme determination

For the extraction of the proteins, 5 g of fresh leaves were ground in liquid nitrogen. The mixed solution was prepared by adding 9 mL protein extraction buffer to the leaf extract. The mixture was centrifuged at 14,000 rpm for 15 min at 4 °C and the supernatants were collected to analyze the total protein content using bovine serum albumin (BSA) as a standard [60].

Superoxide dismutases (SOD) content was measured according to the protocol through [61], followed by inhibition of photochemical reduction with nitroblue tetrazolium (NBT) [62]. A 3 mL assay mixture was prepared from 50 mM phosphate buffer (pH 7.8), 750 µM NBT, 1 µM EDTA, 26 mM L-methionine, 20 µM riboflavin and 100 μL enzyme extract. The SOD content was determined at a wavelength of 560 nm.

The content of catalase (CAT) was determined according to the standard protocol [63] and [5]. The reaction mixture for CAT determination was prepared from 2.8 mL potassium phosphate buffer (25 mM), 100 µL H_2_O_2_ (30 mM) and 100 µL enzyme extract. CAT activity was measured at a wavelength of 240 nm using a spectrophotometer. The enzyme peroxidase (POD) was determined in the spinch plant according to the method [64], with a slight modification of [65, 66]. A mixture was prepared from 100 µL enzyme extract, 2.7 mL potassium phosphate buffer (pH 7.0), 0.1 mL guaiacol (1.5%) and 0.1 mL H_2_O_2_ (0.4%). The POD activity was determined at 470 nm using distilled water as a blank. Ascorbate peroxidase (APX) activity was determined according to the standard protocol [67] with a minor modification of [68]. The reaction mixture for the determination of AP*X* activity was prepared from 100 mM phosphate buffer (pH 7.8), 0.3 mM ascorbic acid, 0.1 mM Na-EDTA, 0.06 mM H_2_O_2_ and 100 µL enzyme extract. APX activity was measured at a wavelength of 290 nm.

#### Expression of spinach ascorbate peroxidase (APX) isozymes genes

RNA standard isolation kit (NucleoSpin RNA plant, Germany) was used to isolate RNA from soil-grown spinach leaves contaminated with heavy metals. The cDNA synthesis kit (Thermo scientific RevertAid First strand) was used to synthesized cDNA using oligo (dT) primers. The stomatal and thylakoid ascorbate peroxidase (*sAPX*, *tAPX*) genes were amplified using primers in the thermocycler (APPLIED BIO SYSTEMS) together with the positive control (actin gene RAc1 and GAPDH). PCR was performed under optimized standard conditions for amplification, e.g.at 95 °C for 5 min for pre denaturation; 30 cycles at 95 °C for 20 Sec, at 60 °C for 30 Sec, at 72 °C for 40 Sec and a final extension for 12 min at 72 °C. The gel documentation system was run on a 1.5% agarose gel to visualize the PCR products.

### Leaf stomata microstructural studies

The scanning electron microscope (SEM) was used for the microstructural examination of the stomata of the leaves according to the manufacturing protocol. Dried spinach leaves were used for the chemical fixation process. The leaf with a size of almost 1-mm2 (*n* = 3) was attached to an aluminum rod using silver paste and placed in the rod holder under vacuum. The sample was examined in the SEM (JEOL JSM–5910, Japan) at a magnification of 4000X and 5 kV.

### Statistical analysis

A one-way analysis of variance (ANOVA) and an LSD test (least significant difference) test were performed to compare the applied for comparison of treatments. A *p* ≤ 0.05 was considered statistically significant.

## Results

### Physiological parameters

#### Germination percentage

**Fig.1:**
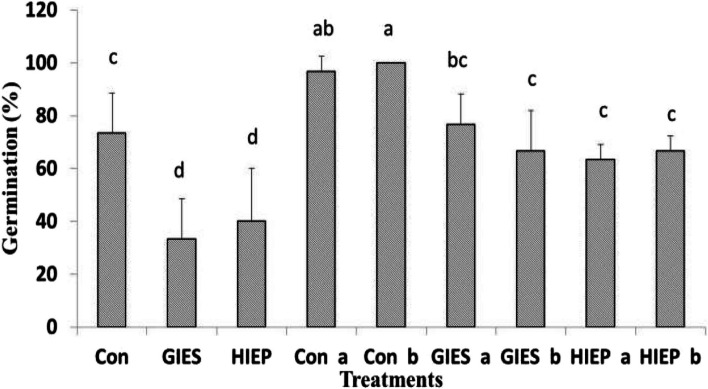
Effects of *Bacillus aerius* (**a**) and *Bacillus cereus* (**b**) on germination (%) of spinach grown in soils contaminated with heavy metals in Gadoon Industrial estate (GIES) and Hayatabad Industrial Estate (HIEP)

The germination behaviour of the spinach seeds differed significantly between the control seeds and the bacterially primed seeds (Fig. 1). The germination of spinach seeds from GIES and HIEP contaminated soils was significantly reduced compared to the control. However, seeds primed with *B. aerius* and *B. cereus* showed improved germination than control. Spinach seed primed with *B. aerius* and *B. cereus* germinated 100% in the control soil, while germination of seeds primed with distilled water was 76%. Similarly, seeds inoculated with *B. aerius* germinated 80% and 72% in GIES and HIEP soil, respectively, while seeds inoculated with *B. cereus* germinated 70% and 72% in GIES and HIEP soil, respectively. Non-inoculated seeds germinated at 30 and 40% in GIES and HIEP soil, respectively (Fig. 1).

#### Plant growth

**Fig. 2:**
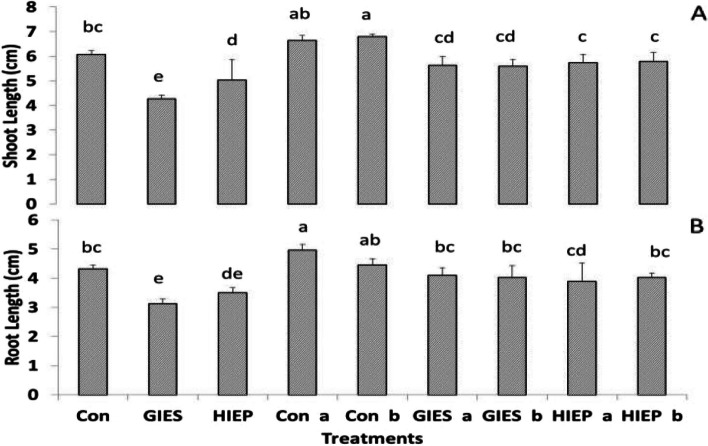
Effect of *Bacillus aerius* (a) and *Bacillus cereus* (b) on shoot (A) and root (B) length (cm/plant) of spinach growing in heavy metal contaminated soils of Gadoon Industrial Estate (GIES) and Hayatabad Industrial Estate (HIEP)

The presence of heavy metals in the soil significantly reduced the seedling length of spinach (Fig. 2). The spinach grown in the control group has a root and shoot length of 4.33 and 6.06 cm/plant respectively. The root and shoot length of spinach plants grown in GIES and HIEP contaminated soil were 3.1 and 4.1 cm and 3.5 and 5.03 cm, respectively (Fig. 2). The root and shoot length of spinach plants grown in control soil inoculated with *B. aerius* and *B. cereus* were 5.2 and 6.7 cm, respectively. The root and shoot length of spinach plants grown in GIES-contaminated soil and inoculated with *B. aerius* were 4.1 and 5.6 cm, respectively, while the root/shoot length of plants inoculated with *B. cereus* were 4.03 and 5.6 cm, respectively. Similarly, the root/shoot length of plants grown with HIEP soil and inoculated with *B. aerius* were 3.9 and 5.7 cm, respectively while the root/shoot length of plants inoculated with *B. cereus* were 4.03 and 5.8 cm, respectively (Fig. 2).

**Fig. 3:**
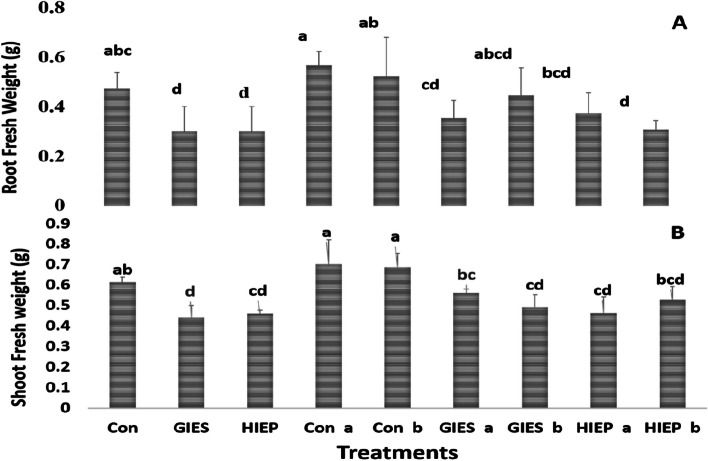
Effect of *Bacillus aerius* (a) and *Bacillus cereus* (b) on the fresh weight of roots (A) and shoots (B) of spinach growing in soils contaminated with heavy metals in the Gadoon Industrial Estate (GIES) and Hayatabad Industrial Estate (HIEP)

The fresh weight of roots and shoots was also significantly altered in spinach plants grown on GIES and HIEP contaminated soils (Fig. 3A and B). The fresh weight of root/shoot of spinach plants grown in control soil was 0.47 and 0.61 g/plant, respectively, while the fresh weight of root/shoot of spinach plants grown in GIES- and HIEP-contaminated soil decreased to 0.30 and 0.44 g/plant and 0.3 and 0.46 g/plant, respectively (Fig. 3A and B). However, the fresh weight of spinach plants inoculated with bacterial strains was significantly higher than that of plants in contaminated soil. Plants grown in GIES contaminated soil and inoculated with *B. aerius* had a significantly higher root/shoot fresh weight of 0.35 and 0.56 g/plant, respectively, while the root/shoot fresh weight of plants inoculated with *B. cereus* was 0.44 and 0.49 g/plant, respectively. In HIEP soil, the fresh weight of spinach roots and shoots was 0.37 and 0.46 g/plant when inoculated with *B. aerius* and 0.30 and 0.52 g/plant when inoculated with *B. cereus* (Fig. 3).

**Fig. 4:**
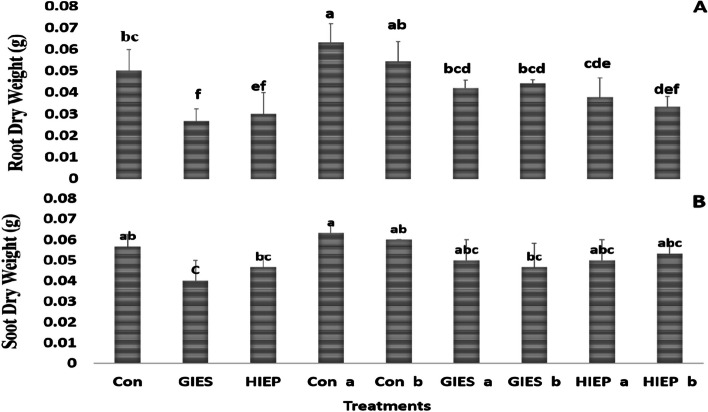
Effect of *Bacillus aerius* (a) and *Bacillus cereus (*b) on the dry weight of roots (A) and shoots (B) dry weight (g/plant) of spinach grown in soil contaminated with heavy metals in the Gadoon Industrial Estate (GIES) and Hayatabad Industrial Estate (HIEP)

Root and shoot dry weights of spinach plants grown in controlled, GIES and HIEP contaminated soil were reported as 0.05, 0.053, 0.02, 0.04, 0.03, and 0.04 g/plant, respectively (Fig. 4). The root/shoot dry weight of spinach plants inoculated with *B. aerius* in GIES contaminated soil was 0.042 and 0.05 g/plant while the root/shoot dry weight of plants inoculated with *B. cereus* was 0.04 and 0.05 g/plant. The root/shoot dry weight of plants inoculated with *B. aerius* in HIEP-contaminated soils was 0.037 and 0.05 g, respectively, while the root/shoot dry weight of plants inoculated with *B. cereus* was 0.33 and 0.53 g/plant, respectively (Fig. 4).

### Biochemical parameters

#### Determination of total nitrogen and protein contents in spinach

**Fig. 5:**
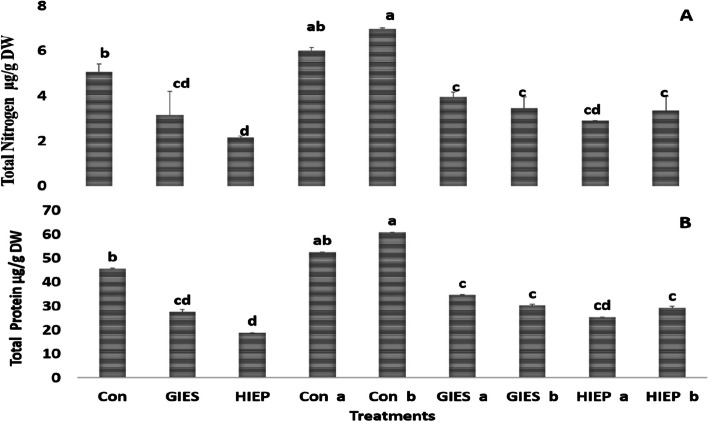
Effect of *Bacillus aerius* (a) and *Bacillus cereus* (b) on total nitrogen (A) and protein contents (B) (µg/g) content of spinach grown in heavy metal contaminated soils of Gadoon Industrial Estate (GIES) and Hayatabad Industrial Estate (HIEP)

In GIESand HIEP polluted soils, spinach plants grew with a nitrogen and protein content of 4.4, 27.57 µg/g and 3.01, 18.82 µg/g, respectively (Fig. 5). Higher total nitrogen and protein contents were analyzed in spinach plants inoculated with bacterial strains. Total nitrogen and protein levels were 5.5 and 4.8 µg/g and 34.57 and 30.20 µg/g in spinach inoculated with *B. aerius* and *B. cereus* strains, respectively. Similarly, total nitrogen and protein levels in HIEP soil were 4.06, 25.38 µg/g when inoculated with *B. aerius* and 4.69, 29.32 µg/g when inoculated with *B. cereus* (Fig. 5).

#### Mathalothionins (MTs) concentration

**Fig. 6:**
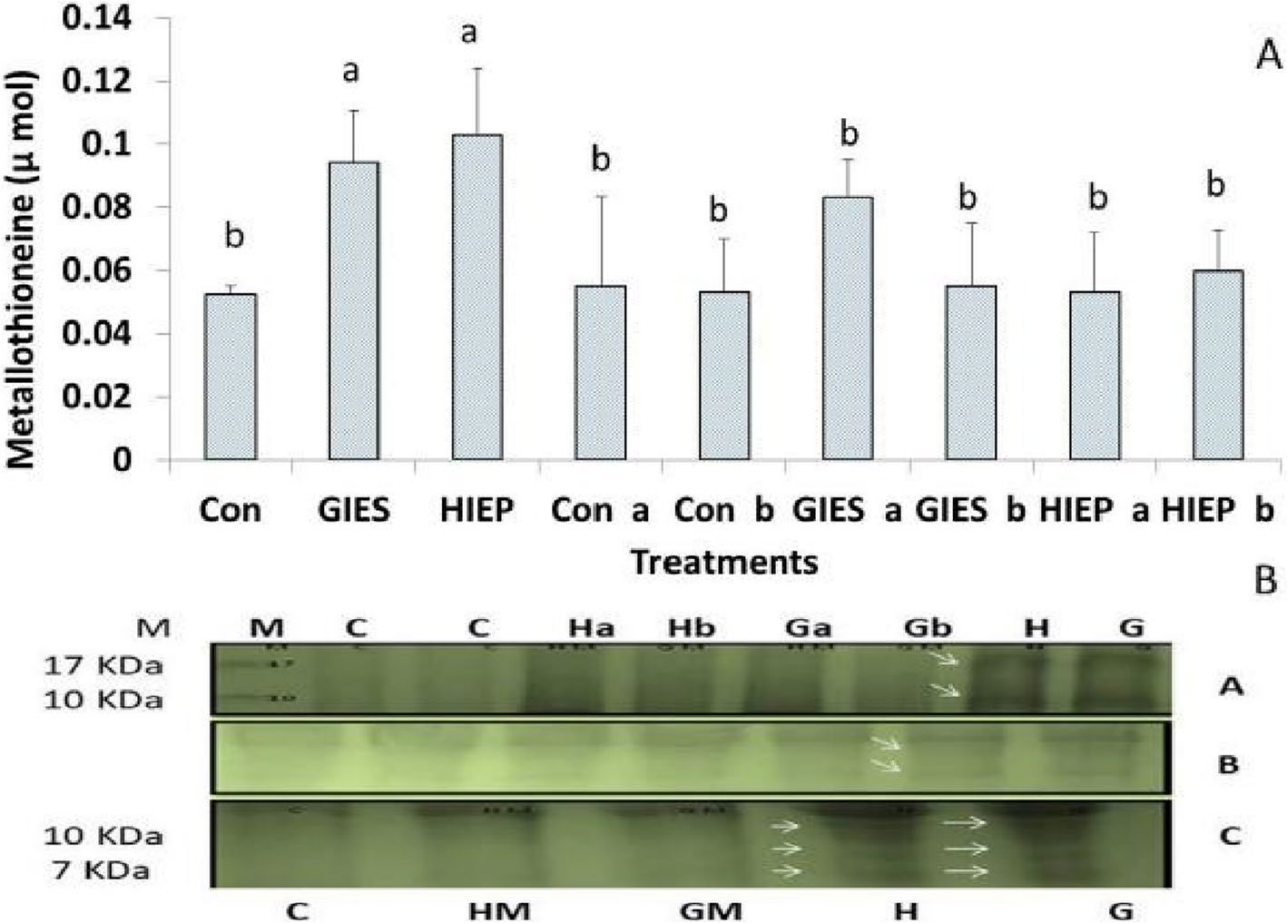
**A**, **B** Effect of *Bacillus aerius* (a) and *Bacillus cereus* (b) on the metallothionein (MTs) of spinach growing in the heavy metal contaminated soils of Gadoon Industrial Estate (GIES) and Hayatabad Industrial Estate (HIEP)

MTs concentrations were higher in spinach plants grown in GIES and HIES contaminated soil. The results showed that MTs concentrations were 0.094, 0.10 and 0.05 µmol in plants grown in GIES, HIEP contaminated soil and control soil, respectively. However, the amount of MTs was lower in spinach plants grown from seeds primed with bacterial strains in GIES and HIEP contaminated soil. The MTs concentrations were 0.083 and 0.053 µmol in spinach plants inoculated with *B. aerius* in GIES and HIEP soil, respectively. Similarly, spinach plants inoculated with *B. cereus* in GIES and HIEP soil had 0.05 and 0.06 µmol MTs concentrations (Fig. 6A). SDS-PAGE electropherograms were used to visualize low molecular weight MTs, which showed 5–17 KDa distinct polypeptide bands on the gel (Fig. 6B). Plants grown on GIES and HIEP contaminated soil showed increased band density, while plants inoculated with microbes did not show responsible polypeptides on the gel.

#### Antioxidative enzymes activities

**Fig. 7:**
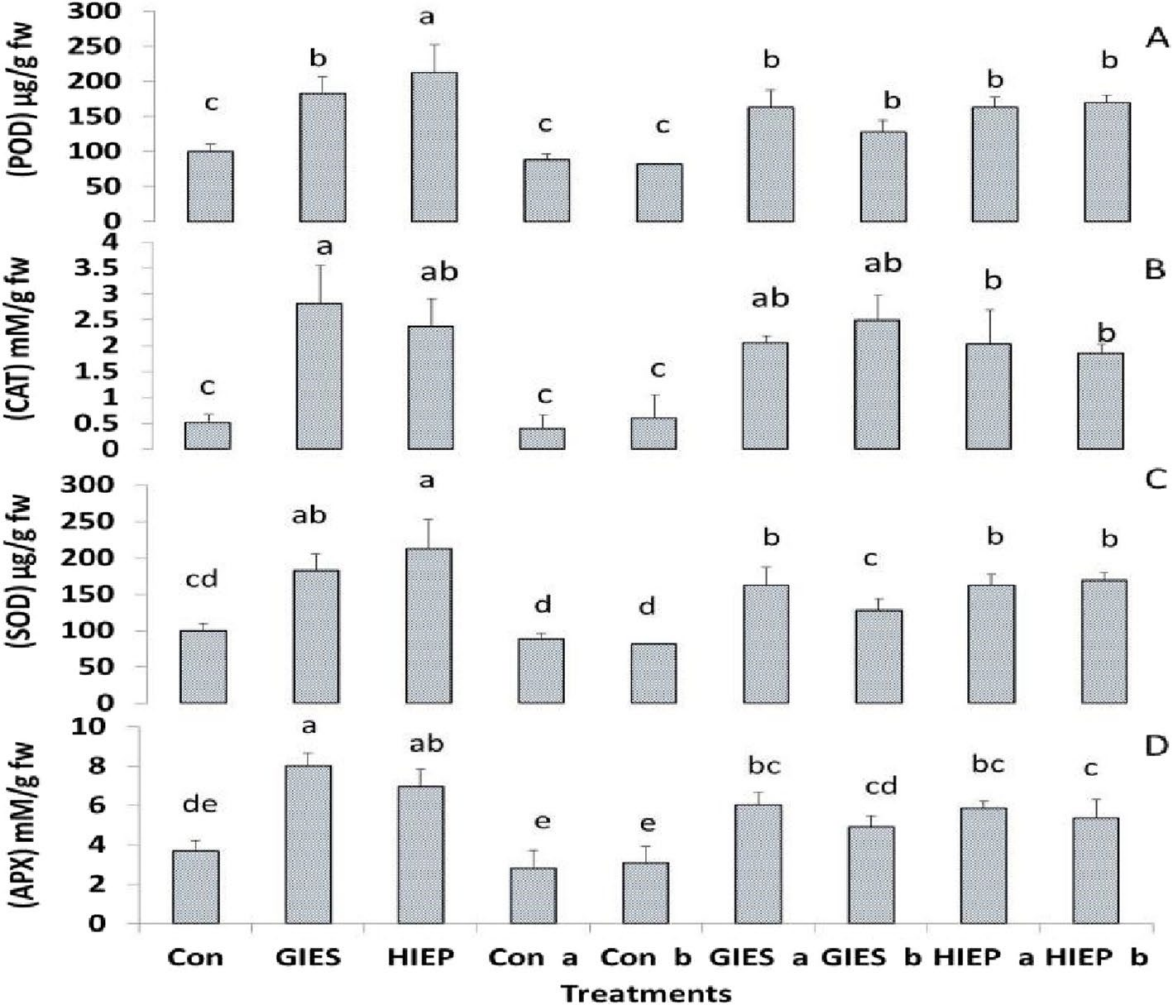
Effect of *Bacillus aerius* (a) and *Bacillus cereus* (b) on antioxidant metabolism (MTs) of spinach grown in soils contaminated with heavy metals in Gadoon Industrial Estate (GIES) and Hayatabad Industrial Estate (HIEP)

The activity of antioxidant enzymes was higher in spinach plants in GIES and HIEP contaminated soil (Fig. 7). The SOD concentrations were 152.59 and 212.05 µg/g FW in the GIES and HIEP plants, respectively, while they were 99.65 µg/g FW in the control plants. The activities of POD, CAT and AP*X* were also 50.06, 2.81, 8.0 µg/g FW and 65.50, 0.53, 0.90 µg/g FW in spinach plants grown on GIES and HIEPcontaminated soils, respectively. A spinach plant grown on control soils had POD, CAT and AP*X* activities of 2.26, 0.50 and 3.66 µg/g FW, respectively. Plants grown from seeds inoculated with *B. aerius* show a significant decrease in antioxidant enzyme activities in GIES and HIEP soils, i.e. SOD (162.01, 161.98 µg/g FW), POD (49.26, 48.90 µg/g FW), CAT (2.05, 2.03 mM/g FW) and AP*X* (6.01, 5.83 mM/g FW) respectively. The same trends in antioxidant enzyme activities were observed when *B. cereus* bacteria were inoculated into GIES and HIEP soil spinach plants (Fig. 7).

#### Ascorbate peroxidases (APX) gene expression in stomata and thylakoids

**Fig. 8:**
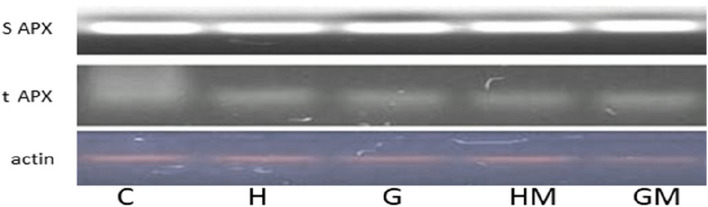
Effects of *Bacillus aerius* (**a**) and *Bacillus cereus* (**b**) on the expression of isoenzymes of chloroplastic ascorbate peroxidase grown in soil contaminated with heavy metals in Gadoon Industrial Estate (GIES) and Hayatabad Industrial Estate (HIEP) C: Control; G: Gadoon Industrial Estate; H: Hayatabad Industrial Estate; M: bacterial strain

In spinach Spinach plants grown on GIES and HIEP soils, the expression of stroma and thylakoid ascorbate peroxidase genes did not change compared to the control. Both ascorbate peroxidase isozymes showed the same expression in spinach leaves after gene specific PCR in all treatments (Fig. 8).

#### Ultramorpological changes in stomata

**Fig. 9:**
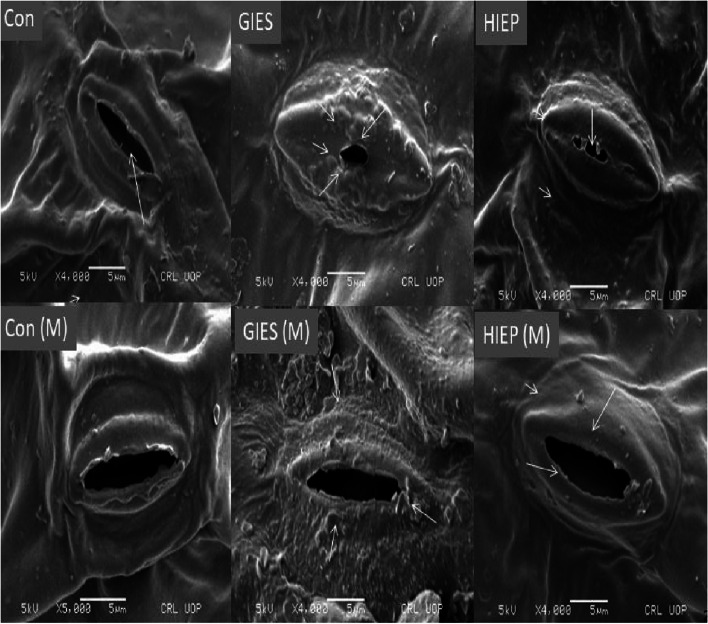
Effect of *Bacillus aerius* (**a**) and *Bacillus cereus* (**b**) on stomatal aperture of spinach grown in soils contaminated with heavy metals in Gadoon Industrial Estate (GIES) and Hayatabad Industrial Estate (HIEP)

The abaxial surface of plant leaves grown on GIES, HIEP and control soils was used to examine the stomata with a scanning electron microscope. It was found that the stomata aperture was close and small in spinach leaves grown on GIES and HIEP contaminated soils In contrast, the stomata of plants grown on *B. cereus* and *B. aerius* inoculated GIES and HIEP soils were open stomatal aperture (Fig. 9).

## Discussion

Plants confronted with heavy metal toxicity have survival, growth and metabolic problems [69]. Plants use numerous techniques to reduce the toxicity of heavy metals or to minimize their entry into plants [70, 71]. However, the use of resistant bacterial strains against heavy metals is an attractive technique as it is nature-friendly, economical and easy to apply [72–76]. In the current study, the physiological and biochemical properties of spinach grown in heavy metal contaminated soils were determined using *B. aerius* and *B. cereus* bacterial strains isolated from heavy metal contaminated soils. In vegetable crops, the most common problems of heavy metal toxicity are low germination rate, slow early growth, reduction in plant biomass, poor metabolism and structural changes in stomata [2, 44, 77, 78]. GIES and HIEP plants contaminated with industrial pollutants showed low germination rate (%) and low fresh seedling biomass (Figs. 1, 2 A, B, 3 A, B, 4 A, B). The literature shows that crops contaminated with heavy metals have reduced seed germination and biomass [2, 79, 80]. The reduction in germination and plant biomass may be due to low water potential, poor nutrient uptake, production of ROS, irregular arrangement of microtubules and suppression of the cell elongation process [81, 82]. The interaction between plants and microbes promotes plant growth and increases tolerance to soils contaminated with heavy metals [83]. Our results showed that plants grown from seeds inoculated with *B. aerius* and *B. cereus* grew better in soils contaminated with heavy metals than in control, GIES and HIEP soils. This could be due to the unavailability of metals to the plant due to various adaptive strategies of the bacteria, including adsorption or absorption of toxic metals, detoxification of metals, and protection from metal contaminated environment [84]. According to [72], the association of plant and bacterium (Methylobacterium oryzae) reduces the uptake of Cd and Ni into plant roots and thus promotes plant growth in heavy metal contaminated soils. The results of the current study were also confirmed by other scientists who used different bacterial strains to improve plant growth under unfavorable environmental conditions [73, 85, 86].

Spinach grown in soil contaminated with heavy metals has lower nitrogen and protein content (Fig. 5A and B). Metal-stressed plants have low protein and nitrogen contents, possibly due to a slowdown in protein synthesis, nitrogen metabolism and upregulation of protease activity [87, 88]. Spinach seeds inoculated with *B. aerius* and *B. cereus* showed higher total nitrogen and protein content. Bacterial strains could reduce the mobility of toxic metals in the soil or block the entry channels of metals into the root system of plants, thereby protecting the important metabolic organelles from the negative effects of metal stress. Plant root bacteria have the potential to improve the metabolic activities of plants in a polluted environment by reducing abiotic stress [89]. These halophilic bacteria increased nitrogen and protein content by providing various nutrients, producing phytohormones, solubilizing of beneficial ions and regulating ACC deaminase activity [86, 90].

The production of MTs in plants against abiotic stress is a well-known phenomenon [78]. High MTs contents were observed in industrial contaminated soil grown plants compared to control (Fig. 6A and B).The current results and previous findings show that MTs production correlates with metal stress in plants [91]. SDS-PAGE profiling also confirmed these results by showing high intensity polypeptide bands between 5–18 KDa. Abdelmigid et al. [92] also demonstrated that 7–17 KDa polypeptide MTs bands were expressed in *Brassica* under Cd induced stress. Reduced MTs expression was observed in spinach seedlings grown in polluted soil from seeds treated with two bacterial strains. This low level of MTs in the plants also suggests that the two bacterial strains help in the removal of heavy metal stress. Secondly, *B. aerius* and *B. cereus* can also prevent the transport of toxic metals to the plant organs [44].

Reactive oxygen species are produced in stressed plants [93]. Under stress conditions, the immune system of plants activates antioxidant enzymes to scavenge ROS [94, 95]. ROS production is increased in plants in a medium contaminated with heavy metals, which is in proportion to the production of antioxidants [96]. Current research confirms that spinach plants activate antioxidant enzymes against the heavy metals present in the soil to cope with the stress conditions. In contrast, plants grown in contaminated soils inoculated with *B. aerius* and *B. cereus* bacterial strains showed lower levels of antioxidant enzymes (Fig. 7). The addition of heavy metals to soil causes plant toxicity, but the presence of soil bacteria mitigates the negative effects of heavy metals on plant growth [97, 98]. In polluted ecosystems, microbes improve plant growth by reducing the toxicity of metals through various mechanisms such as immobilization, complexation, alkalinization, transformation, precipitation, or chelation of toxic metals [74, 99]. All these mechanisms reduce or slow down the production of ROS in plants because they block the plant root channels for the entry of heavy metals [99]. The expression profile of tAPX and sAPX showed the same expression in plants grown on soil contaminated with heavy metals (Fig. 8). According to [100], *pre*-mRNA splicing in the chloroplast of spinach. These isoenzymes (genes) are constitutively expressed to protect the plant from ROS stimulated photo-oxidative stress [101].

Stomata closing mechanisms allow plants to prevent the transpiration process, as heavy metals prevent water uptake by plant roots [102–105]. The stomata activity of the spinach plant is significantly impaired by heavy metals in polluted soils (Fig. 9). Heavy metal induced toxicity affected the shape and size of stomata in plants grown in soil polluted with industrial areas compared to bacterially primed plants (Fig. 9). Similar results were observed by [106], they reported the closure of stomata in *Helianthus annulus* when treated with effluents from the tannery industry. Heavy metal stress inhibits the water supply to the stomata and thus reduces the size of the stomata aperture [106, 107]. Moreover, the stomata were open in the plant leaves inoculated with microbes, indicating a protective role in reducing the toxicity of heavy metals. The microbes stop the movement of heavy metals to the plant organs and mitigating the stress effect on the opening and closing of the stomata aperture [44].

## Conclusions

The conclusion is that heavy metals significantly impair plant growth by inducing oxidative stress in the spinach plant. However, *B. aerius* and *B. cereus* improved the physiological and biochemical parameters of plants grown in soils contaminated with heavy metals by reducing soil toxicity caused by the deposition of heavy metals and normalizing oxidative stress by reducing the synthesis of ROS. In addition, plants inoculated with both bacterial strains had greater stomata aperture as compared to untreated plants. It can be concluded that the bacterial strains improved plant growth by increasing the rate of photosynthesis and reducing the uptake of heavy metals from the polluted soil.

### Supplementary Information

**Additional file 1:** **Fig. S1.** Effect of *Bacillus aerius* (a) and *Bacillus cereus* (b) on the metallothionein (MTs) of spinach growing in the heavy metal contaminated soils. Effects of *Bacillus aerius* (c) and *Bacillus cereus* (d) on the expression of isoenzymes of chloroplastic ascorbate peroxidase grown in soil contaminated with heavy metals.

## Data Availability

All data generated or analysed during this study are included in this published article. The Raw images of the gel/blot were included in Supplementary Data file (Fig. S1).

## Abbreviations

ROS: Reactive oxygen species
SOD: Superoxide dismutases
CAT: Catalase
POD: Peroxidase
APX: Ascorbate peroxidase
Con: Control
Con a: Control with *Bacillus aerius*
Con b: Control with *Bacillus cereus*
GIES: Gadoon ındustrial estate
GIES a: Gadoon ındustrial estate *Bacillus aerius*
GIES b: Gadoon ındustrial estate *Bacillus cereus*
HIEP: Hayatabad ındustrial estate
HIEP a: Hayatabad ındustrial estate with *Bacillus aerius*
HIEP b: Hayatabad ındustrial estate with *Bacillus cereus*
