# Supplementary material for: Inoculation of heavy metal resistant bacteria alleviated heavy metal-induced oxidative stress biomarkers in spinach (Spinacia oleracea L.)
Source: BMC Plant Biol. 2024 Mar 27;24:221. doi: 10.1186/s12870-024-04757-7 (PMC10976752; doi:10.1186/s12870-024-04757-7)
Supplement: Supplementary file 1 — Additional file 1: Fig. S1. Effect of Bacillus aerius (a) and Bacillus cereus (b) on the metallothionein (MTs) of spinach growing in the heavy metal contaminated soils. Effects of Bacillus aerius (c) and Bacillus cereus (d) on the expression of isoenzymes of chloroplastic ascorbate peroxidase grown in soil contaminated with heavy metals. [file 12870_2024_4757_MOESM1_ESM.docx]

**Supplementary Data**

**(a)
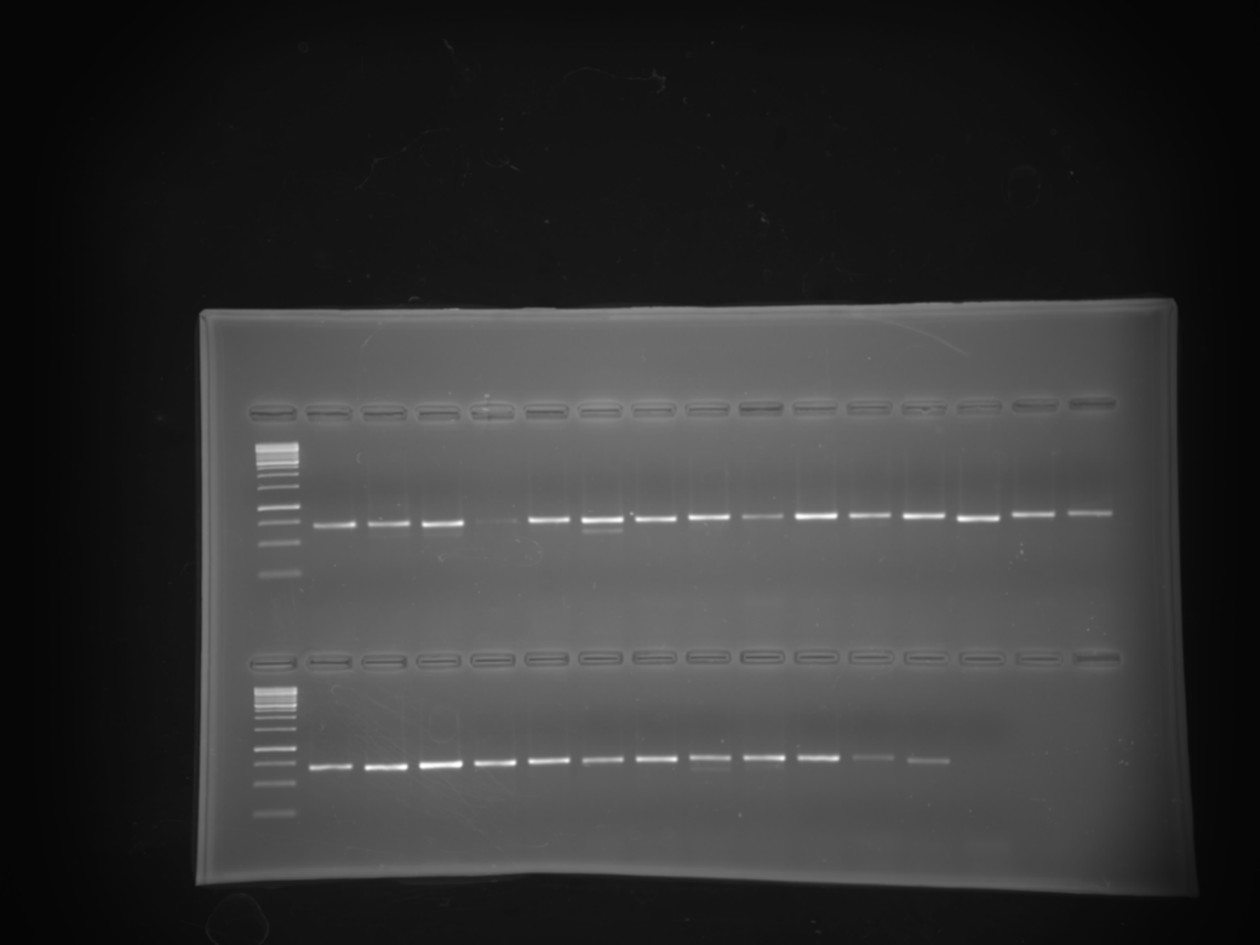
**

**(b)
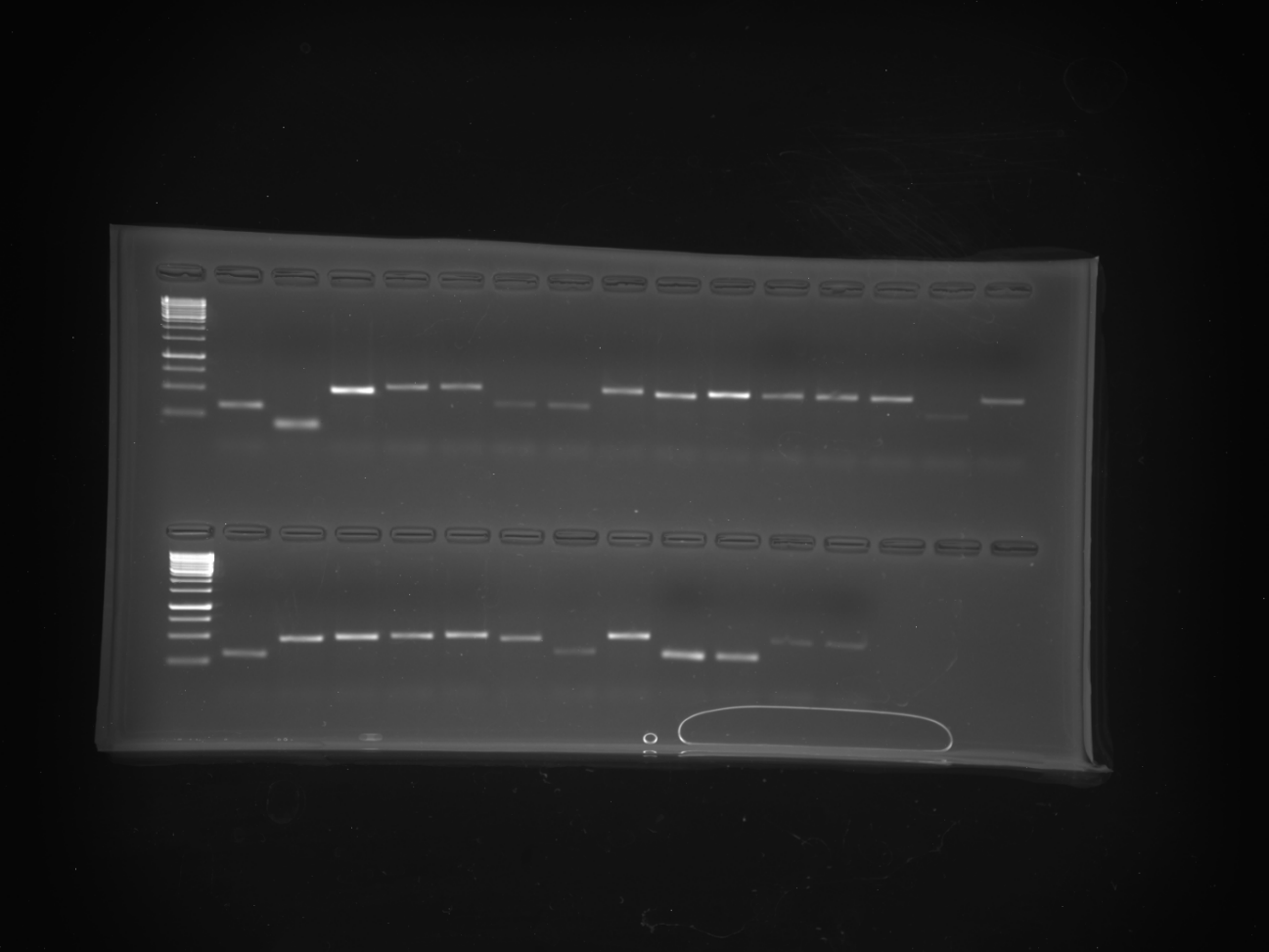
**

**(c)
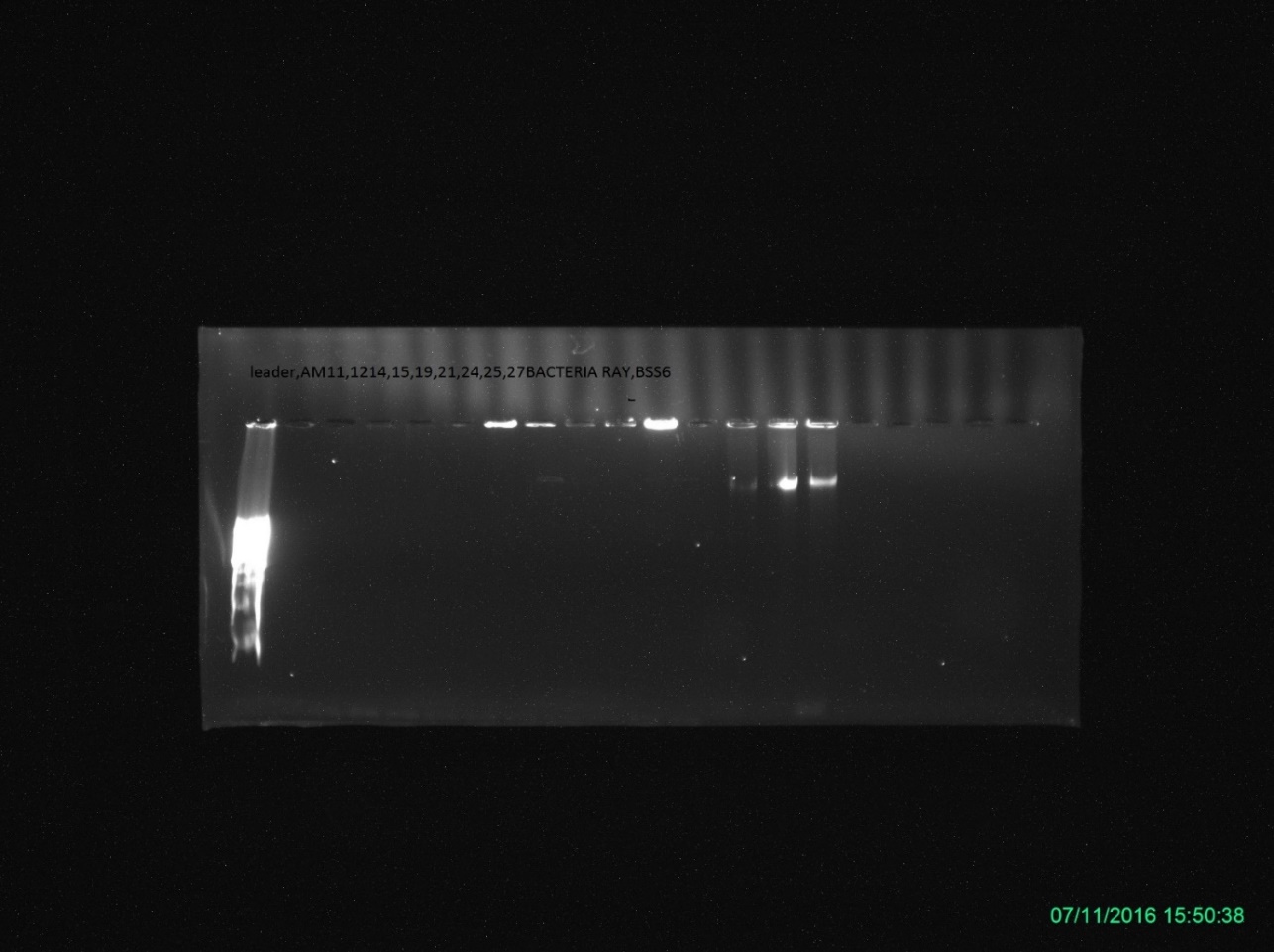
**

**(d)
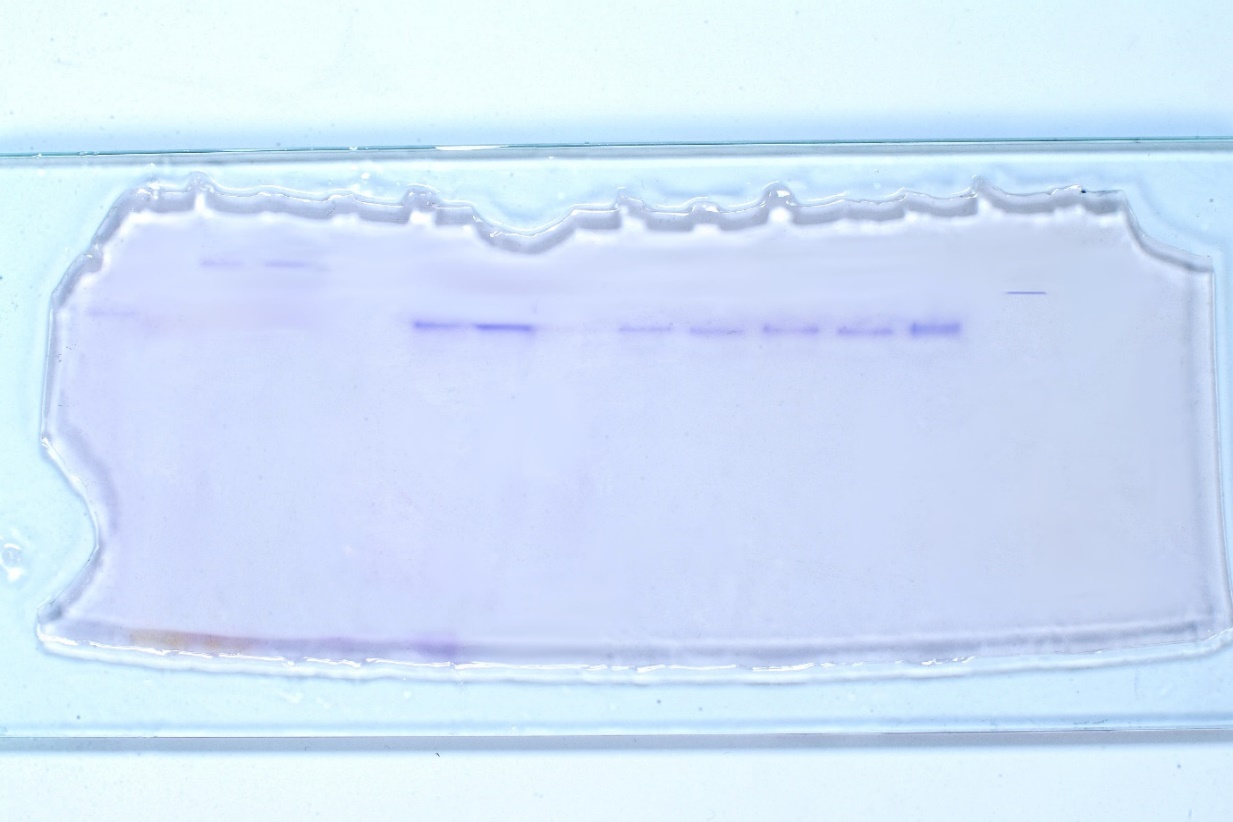
**

**Fig S1.** Effect of *Bacillus aerius* (a) and *Bacillus cereus* (b) on the metallothionein (MTs) of spinach growing in the heavy metal contaminated soils. Effects of *Bacillus aerius* (c) and *Bacillus cereus* (d) on the expression of isoenzymes of chloroplastic ascorbate peroxidase grown in soil contaminated with heavy metals.
